# Removal of Cholera Toxin from Aqueous Solution by Probiotic Bacteria

**DOI:** 10.3390/ph5060665

**Published:** 2012-06-19

**Authors:** Jari E. Heikkilä, Sonja M. K. Nybom, Seppo J. Salminen, Jussi A. O. Meriluoto

**Affiliations:** 1 Department of Biosciences, Biochemistry, Åbo Akademi University, Tykistökatu 6A, 20520 Turku, Finland; 2 Functional Foods Forum, University of Turku, 20014 Turku, Finland

**Keywords:** cholera toxin, probiotic bacteria, *Lactobacillus*, *Bifidobacterium*

## Abstract

Cholera remains a serious health problem, especially in developing countries where basic hygiene standards are not met. The symptoms of cholera are caused by cholera toxin, an enterotoxin, which is produced by the bacterium *Vibrio cholerae*. We have recently shown that human probiotic bacteria are capable of removing cyanobacterial toxins from aqueous solutions. In the present study we investigate the ability of the human probiotic bacteria, *Lactobacillus rhamnosus* strain GG (ATCC 53103) and *Bifidobacteriumlongum* 46 (DSM 14583), to remove cholera toxin from solution *in vitro*. *Lactobacillus rhamnosus* strain GG and *Bifidobacteriumlongum* 46 were able to remove 68% and 59% of cholera toxin from aqueous solutions during 18 h of incubation at 37 °C, respectively. The effect was dependent on bacterial concentration and *L. rhamnosus *GG was more effective at lower bacterial concentrations. No significant effect on cholera toxin concentration was observed when nonviable bacteria or bacterial supernatant was used.

## 1. Introduction

Cholera is an infectious acute diarrhoeal disease caused by the bacterium *Vibrio cholerae.* If left untreated cholera can be fatal, leading to rapid death within few hours. Despite efforts made to prevent and control cholera, outbreaks of cholera are still common and according to WHO estimations, 120,000 people die annually from the disease [1], most of the victims being children [2]. An oral killed whole-cell B-subunit vaccine is available [3], but it needs to be administered twice at 10–14 day intervals and children aged two to six years need three doses. In addition, the vaccine must be taken with a large volume of pure liquid and requires cold chain distribution [1] and the cost in some cases is prohibitive, so novel means of fighting the disease are urgently needed. *Vibrio cholerae* produces an enterotoxin, cholera toxin, which is responsible for the diarrhoeal symptoms [4]. Cholera toxin is composed of five inactive B-subunits, which bind ganglioside GM1 receptors of the intestinal mucosal cells and allow the catalytically active A-subunit to enter the mucosal cells [5]. The A-subunit ribosylates regulatory G-protein leading to activation of adenylate cyclase and increased cellular cAMP [6], which in turn stimulates mucosal cells to pump large amounts of Cl^−^ into the intestine followed by Na^+^ and water [7].

Probiotics are defined as live microbial food supplements which have a beneficial effect on human health when taken in right concentrations [8]. Currently used probiotics have a long history of safe use in food and food fermentations and they are recognized as safe for human consumption. A large number of studies show that probiotics have therapeutic benefits [9]. In particular, probiotics have been reported to reduce the incidence of three kinds of diarrhoea: antibiotic-associated [10], community acquired [11] and infectious, including rotavirus-associated [12]. In addition, duration of gastroenteritis in infants was significantly reduced after treatment with probiotics [13]. Also, probiotics have been shown to reduce disease activity in patients suffering of inflammatory bowel disease [14]. The protective role of probiotic bacteria against gastrointestinal pathogens is not restricted to microbes. Several studies indicate that probiotics can bind toxins such as aflatoxins [15], ochratoxin A [16] and Shiga toxin [17]. Recently, we have shown that probiotics can eliminate the cyanobacterial toxin microcystin-LR from solution [18,19,20]. The aim of the present study was to asses the potential of probiotics to eliminate cholera toxin from aqueous solution.

## 2. Experimental

### 2.1. Chemicals

Acetonitrile (HPLC S-grade) was from Rathburn (Walkerburn, UK), trifluoroacetic acid (TFA) was from Fluka (Buchs, Switzerland) and formic acid (analytical-reagent grade) was from Riedel-de Haën (Seelze, Germany). Water was purified to 18.2 MΩcm on a Milli-Q Synthesis system (Millipore, Molsheim, France).

### 2.2. Cholera Toxin

Cholera toxin (Sigma-Aldrich, St. Louis, MO, USA) was reconstituted in water at 1 mg/mL.

### 2.3. HPLC and LC-MS Analysis

**Figure 1:**
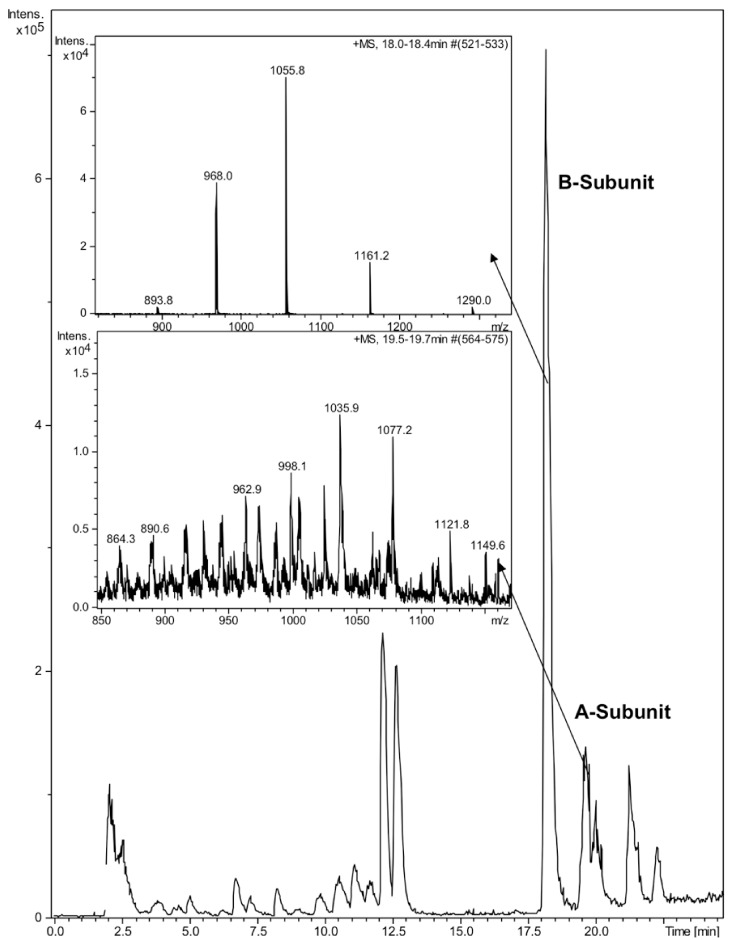
LC-MS analysis of cholera toxin. 10 μL of CTX (100 ng/μL) was run on a Discovery Bio C5 column. Enlarged ion trap MS spectra of the A- and B-subunits of CTX.

CTX was quantified by reverse phase high-performance liquid chromatography (HPLC) on a Discovery Bio C5 column (50 × 4.6 mm, 3 μm particles) with a UV detector at 214 nm. The mobile phase consisted of a gradient of 0.05% aqueous trifluoroacetic acid (solvent A) and 0.05% TFA in acetonitrile (solvent B) with the following linear gradient program: 0 min 5% B, 60 min 90% B, 61 min 5% B. The injection volume was 50 μL, the flow rate 1 mL/min and the column oven temperature 40 °C. The identity of the HPLC peaks was confirmed by ion trap mass spectrometry (Bruker HCT Ultra, Bruker Daltonik GmbH, Bremen, Germany). Due to denaturing nature of the HPLC eluent, no intact holotoxin or pentameric B-subunit could be detected (Figure 1). The mobile phases for ion trap consisted of 0.1% formic acid and acetonitrile. The molecular weights of the subunits 11,602 for the B-subunit and 26,905 for the A-subunit were in a good agreement with previously reported values [21]. Due to the poor sensitivity of detection of the A-subunit by HPLC, we focused on the B-subunit, which was well separated on HPLC and detected at 214 nm.

### 2.4. Bacterial Strains and Cultivation

*L. rhamnosus* GG (ATCC 53103, Valio Ltd., Helsinki, Finland) and *B. longum* 46 (Probiotical srl, Novara, Italy) were grown in MRS broth (Oxoid Ltd, Basingstoke, UK) and MRS broth supplemented with 0.05% cysteine-HCl, respectively, for 18 h at 37 °C in a volume of 50 mL. The bacteria were harvested by centrifugation at 3,200 × *g* for 12 min and washed twice with phosphate-buffered saline (PBS). Finally, the bacteria were resuspended in 4.0 ml PBS at 2–4 × 10^10^ colony forming units (CFU)/mL. The bacterial suspensions were incubated in 1.5 mL borosilicate glass chromatographic vials in a volume of 600 μL together with CTX (24 ng/μL) at 37 °C under continuous shaking. Triplicate samples of 130 μL were removed immediately after addition of CTX (0 sample), after 60 min and after 18 h incubation. The samples were centrifuged at 6,000 × *g* for 5 min in 200 μL borosilicate chromatographic inserts, the resulting supernatants were removed and CTX remaining in solution was determined by HPLC. The number of viable *L. rhamnosus* GG was determined on MRS agar plates in aerobiosis and *B. longum* 46 on MRS agar plates supplemented with cysteine-HCl under anaerobic conditions (Concept 400 anaerobic chamber, Ruskin Technology Ltd., Leeds, UK) after incubation at 37 °C for 48 h.

## 3. Results and Discussion

**Figure 2:**
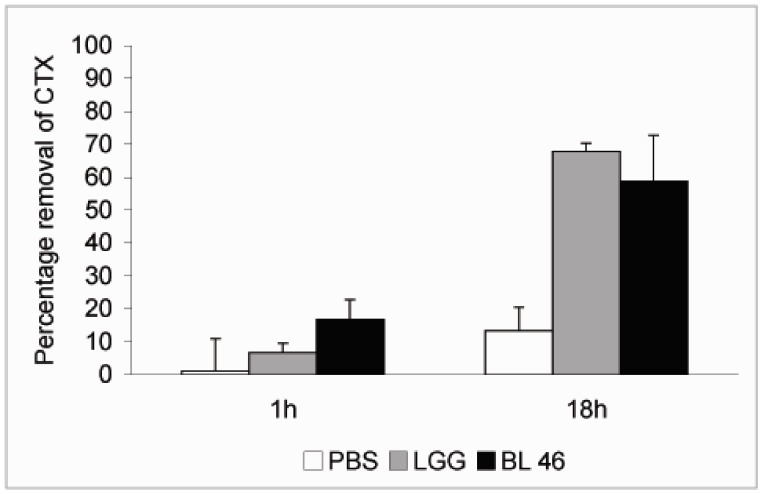
Removal of CTX by probiotic bacteria. CTX (24 ng/μL) was incubated in the presence of PBS, *L. rhamnosus* GG (1.8 × 10^10^ CFU/ml) or *B. longum* 46 (4.0 × 10^10^ CFU/mL) at 37 °C. Samples were removed after 1 and 18 h of incubation and the amount B-subunit of CTX in the supernatant was determined by HPLC.

**Figure 3:**
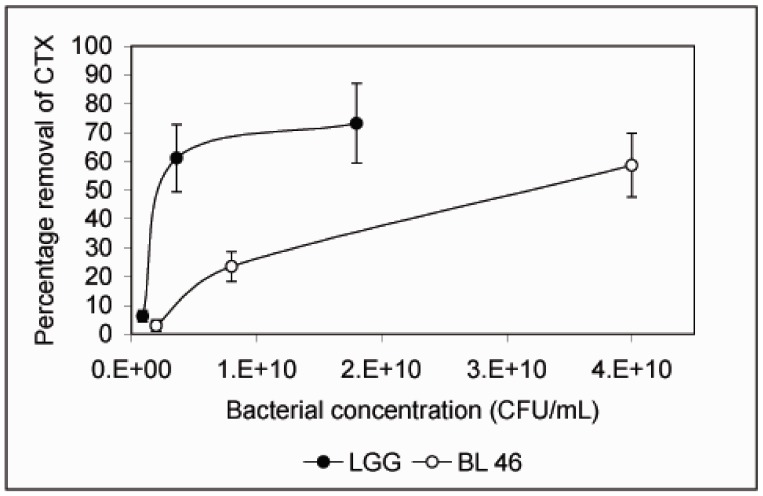
Removal of CTX by *L. rhamnosus* GG and *B. longum* 46 is dependent on the bacterial concentration. CTX (24 ng/μL) was incubated in the presence of *L. rhamnosus* GG (0.09, 0.36 and 1.8 × 10^10^ CFU/mL) or *B. longum* 46 (0.2, 0.8 and 4.0 × 10^10^ CFU/mL) for 18 h and the amount B-subunit of CTX remaining in the supernatant was determined by HPLC.

*L. rhamnosus* GG and *B. longum *46 both caused a significant reduction in the level of CTX during an 18 h incubation. Compared to CTX levels in samples taken immediately after addition of the toxin 68% and 59% of the toxin was removed from solution after incubation with *L. rhamnosus* GG and *B. longum* 46, respectively (Figure 2). The rate of CTX removal appeared to be relatively slow since after one hour of incubation the concentration of the toxin had decreased by 6.6% in the presence of *L. rhamnosus* GG and 16.8% in the presence of *B. longum *46 (Figure 2). In future experiments more time points are though needed to confirm the time dependence of CTX removal. Instability of the toxin in PBS at 37 °C is unlikely since the level of CTX decreased only by 13.3% after 18 h of incubation in samples incubated in PBS alone (Figure 2). When CTX removal from solution was studied as a function of bacterial concentration, *L. rhamnosus* GG was found to be more effective than *B. longum* 46. At 4 × 10^9^ CFU/mL *L. rhamnosus* GG removed 61.1% of CTX during an 18 h incubation whereas *B. longum* 46 only removed 23.5% of CTX at 8 × 10^9^ CFU/mL (Figure 3).

**Figure 4:**
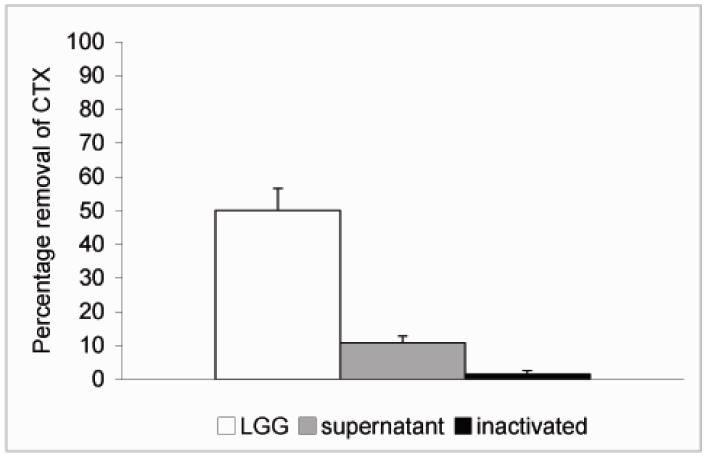
Removal of CTX by *L. rhamnosus* GG cell free supernatant and inactivated bacteria. *L. rhamnosus* GG (4.0 × 10^9^ CFU/mL) was incubated at 37 °C for 6 h and the bacteria were spun down. CTX (24 ng/μL) was added to the supernatant and incubated for 18 h. To study the role of cell viability on the removal of CTX, the bacteria were inactivated by ethanol treatment, resuspended in PBS at 4.0 × 10^9^ CFU/mL and incubated in the presence of CTX (24 ng/μL) for 18 h. In a parallel experiment CTX was incubated in the presence of *L. rhamnosus* GG for 18 h. The amount B-subunit of CTX remaining in solution was determined by HPLC.

In order to study whether cell viability had any effect on the ability of the probiotics to remove CTX from solution *L. rhamnosus* GG was inactivated by treatment with 96% ethanol for 30 min followed by two rounds of washes in PBS. The inactivated bacteria were resuspended in PBS at 4 × 10^9^ CFU/mL and CTX added as described above. No significant reduction in the level of CTX was observed when incubated in the presence of inactivated *L. rhamnosus* GG (Figure 4). We also tried to inactivate the bacteria by heat treatment at 90 °C for 30 min but the results were not consistent due to high variation and therefore we chose to use ethanol inactivation in our experiments. Since it is possible that extracellular proteinases secreted by the bacteria or intracellular proteinases released by dying bacteria are responsible for the effect on CTX observed above, *L. rhamnosus* GG was incubated at 4 × 10^9^ CFU/mL in PBS for 6 h at 37 °C. This incubation time was chosen based on results from our previous study, which showed that removal of microcystin-LR by *L. rhamnosus* GG reached its maximum by 5 h [19]. The bacterial suspension was centrifuged as described above and supernatant removed into another borosilicate vial and CTX (24 ng/μL) was added and incubated at 37 °C for additional 18 h. Only a modest 10.7% reduction in the level of CTX was observed with 89.3% of CTX remaining in solution after incubation in the cell-free supernatant (Figure 4). Thus it is unlikely that secreted or released proteinases would be responsible for the CTX removal.

The effects of *L. rhamnosus* GG and *B. longum* 46 on CTX levels obtained in this study are in agreement with our previous results obtained with microcystin-LR and the two probiotics [18,19]. Both toxins show similar kinetics of removal by the probiotics and viable bacteria were required for effective removal of the toxins. Aflatoxin B_1_, on the other hand, was eliminated extremely rapidly from solution and inactivated bacteria were even more effective than viable ones in removing aflatoxin B_1_ from solution [15,22]. Protein and peptide toxins might thus be removed by a different mechanism than non-protein toxins. Lactobacilli have been shown to utilize cell envelope-associated proteinases to degrade proteins followed by uptake of the resulting oligopeptides by specialized transport systems [23]. This kind of system might also be responsible for the removal of cholera toxin observed in this study.

Recent long-lasting outbreaks of cholera in Africa [24] and the fact that the number of cholera cases is on the rise demonstrate that new approaches are needed to control the disease. Current therapy of cholera is largely based on fluid and electrolyte replacement. Oral rehydration solution (ORS) and in severe cases intravenous fluids are life saving when available. Oral vaccine is also available but requires pure water for administration and cold chain for transport. Since cholera toxin is primarily responsible for the symptoms of cholera, elimination of the toxin in the alimentary tract by probiotic bacteria would prevent the diarrhoeal symptoms. A non-pathogenic strain of *Escherichia coli* engineered to express a mimic of the ganglioside receptor GM_1_ on its surface was recently shown to bind cholera toxin and protect infant mice from *V. cholerae*-induced death [25]. Lactobacilli and bifidobacteria are part of normal intestinal microflora and they have a long history of human consumption and have been proven safe in clinical trials [9,26]. The use of non-engineered probiotics would greatly reduce any possible health risks that might be associated with recombinant bacteria. This would be an advantage especially when treating infants who cannot be given the currently used cholera vaccine and who carry the highest burden of cholera [2]. In addition, antibiotics are part of the recommended cholera treatment regimen [27,28,29] and live probiotics have a well documented history in the treatment of antibiotic-associated diarrhea [10,13] and would not require the use of killed bacteria as might be the case with *E. coli* strains.

## 4. Conclusions

The results from this preliminary study show that both *L. rhamnosus* GG and *B. longum* 46 are capable of eliminating cholera toxin from aqueous solution. Significant toxin removal was observed only with live bacteria. Both strains may have potential in the treatment of cholera, especially in regions where the cold chain of cholera vaccine transportation and/or supply of pure water cannot be guaranteed.
